# Comparative Microbiological and Whole-Genome Analysis of *Staphylococcus aureus* Populations in the Oro-Nasal Cavities, Skin and Diabetic Foot Ulcers of Patients With Type 2 Diabetes Reveals a Possible Oro-Nasal Reservoir for Ulcer Infection

**DOI:** 10.3389/fmicb.2020.00748

**Published:** 2020-04-30

**Authors:** Brenda A. McManus, Blánaid Daly, Ioannis Polyzois, Pauline Wilson, Gráinne I. Brennan, Tanya E. Fleming, Liam D. Grealy, Marie-Louise Healy, David C. Coleman

**Affiliations:** ^1^Microbiology Research Unit, Division of Oral Biosciences, Dublin Dental University Hospital, Trinity College Dublin, The University of Dublin, Dublin, Ireland; ^2^Division of Public and Child Dental Health, Dublin Dental University Hospital, Trinity College Dublin, The University of Dublin, Dublin, Ireland; ^3^Division of Restorative Dentistry and Periodontology, Dublin Dental University Hospital, Trinity College Dublin, The University of Dublin, Dublin, Ireland; ^4^Department of Endocrinology & Diabetes, St. James’s Hospital, Dublin, Ireland; ^5^National MRSA Reference Laboratory, St. James’s Hospital, Dublin, Ireland

**Keywords:** *Staphylococcus*, diabetes, diabetic foot ulcer infection, periodontal disease, nasal carriage, oral carriage

## Abstract

Patients with type 2 diabetes are at higher risk for periodontal disease and diabetic foot ulcer infections (DFUIs), the latter of which are predominantly caused by staphylococcal bacteria. Staphylococci have also been detected in the mouth, nose and gums (the oro-nasal cavity) of patients with periodontal disease and can move between the mouth and nose. The present study investigated if the oro-nasal cavity and/or periodontal pockets (PPs) in diseased gum tissue can provide a microbial reservoir for DFUIs. Eighteen patients with type 2 diabetes and at least three natural teeth (13 patients with ulcers and 5 patients without ulcers) underwent non-invasive microbiological sampling of PP, oro-nasal, skin and ulcer sites. Staphylococci were recovered using selective chromogenic agar, definitively identified and subjected to DNA microarray profiling, whole-genome sequencing and core-genome multilocus sequence typing (cgMLST). *Staphylococcus aureus* and *Staphylococcus epidermidis* were recovered from both the oro-nasal and ulcer sites of 6/13 and 5/13 patients with ulcers, respectively. Molecular typing based on the staphylococcal protein A (*spa*) gene and DNA microarray profiling indicated that for each patient investigated, *S. aureus* strains from oro-nasal and ulcer sites were identical. Comparative cgMLST confirmed that isolates from multiple anatomical sites of each individual investigated grouped into closely related, patient-distinct clusters (Clusters 1–7). Isolates belonging to the same cluster exhibited an average of 2.9 allelic differences (range 0–11). In contrast, reference genomes downloaded from GenBank selected as representatives of each sequence type identified in the present study exhibited an average of 227 allelic differences from the most closely related isolate within each cluster.

## Introduction

Current estimates indicate that 422 million people worldwide and approximately 4.3% of people living in Ireland have diabetes (International Diabetes Federation, 2017). Approximately 90% of these have type 2 diabetes, characterized by insufficient insulin secretion or metabolism, resulting in hyperglycemia.

Periodontal disease (PD) is a polymicrobial disease that is caused by the stimulation of the inflammatory process in response to dental plaque. The risk of PD is two–threefold higher in patients with diabetes due to hyperglycemia of host tissues and reduced salivary flow (Casanova et al., 2014). During PD progression the detachment of gingival tissue from teeth results in the formation of periodontal pockets (PPs) and eventually the loss of underlying support structures.

Diabetic foot ulcer infections (DFUIs) are a potentially disastrous complication of diabetes, significantly increasing the risk of lower extremity amputation. In Ireland, incidences of diabetes-related lower extremity amputations have increased consistently since 2005 (Buckley et al., 2012; Diabetes Ireland, 2015). A DFUI typically begins as a monomicrobial superficial infection which may progress to severe, deep, chronic, and polymicrobial infection, often involving anaerobic bacteria due to the low redox potential of associated tissues (Charles et al., 2015).

The Gram-positive bacterial pathogen *Staphylococcus aureus* is the leading cause of all skin and soft tissue infections including DFUIs and can also cause more severe infections such as necrotizing pneumonia and sepsis. *S. aureus* predominantly colonizes the nares, persistently or transiently, but is also commonly recovered from the mouth, skin, perineum and pharynx. Approximately one-third of people are nasally colonized with this species which presents an important risk factor for potential infection (Wertheim et al., 2005; Krismer et al., 2017; Sakr et al., 2018).

The *S. aureus* genome consists of both a core and accessory genome, the combination of which harbors an impressive arsenal of genes encoding toxins and virulence factors such as leukocidins, hemolysins, and enterotoxins. *S. aureus* is also adept at acquiring antimicrobial resistance genes from other bacterial species, as evidenced by the acquisition of the staphylococcal chromosomal cassette element harboring *mec* (SCC*mec*) and the independent emergence and pandemic success of several clones of methicillin-resistant *S. aureus* (MRSA) (Monecke et al., 2011). Some virulence genes can also be exchanged among *S. aureus* strains as well as between coagulase-negative staphylococcal species (CoNS) and *S. aureus*. It is believed that the acquisition of the arginine catabolic mobile element (ACME) from CoNS is one of the main reasons for the pandemic spread of the USA300 MRSA clone from the United States.

The advent of whole-genome sequencing (WGS) has dramatically improved the discrimination of isolates during outbreak investigations and provides an immensely powerful tool for rapid epidemiological, population structure and antibiotic susceptibility pattern analyses. Conventional molecular typing-based methods such as multilocus sequence typing (MLST) and staphylococcal protein A (*spa*) typing which target seven and one loci, respectively, have largely been replaced by WGS-based methods such as core-genome MLST (cgMLST). Despite these advances, *S. aureus* populations are still commonly described according to the traditional MLST-based sequence type (ST) and *spa* type. However, cgMLST is significantly more discriminatory and the publicly available cgMLST scheme for *S. aureus* investigates 1,861 loci, providing a standardized reference system by which isolates from distinct lineages can be directly compared. The maximum number of allelic differences detected between isolates deemed closely related can vary according to each distinct clonal lineage investigated, as well as the timeframe over which the isolates are recovered. A general consensus guideline of ≤24 allelic differences is deemed to reflect closely related isolates, but ideally relatedness should be considered in the context of available associated epidemiological information (Leopold et al., 2014; Bartels et al., 2015; Schürch et al., 2018).

The link between nasal colonization with *S. aureus* and endogenous infection risk is well documented (Wertheim et al., 2005; Sakr et al., 2018; Gagnaire et al., 2019). Researchers have also reported the recovery of identical *S. aureus* isolates from the nares and ulcers of 65% of 276 patients with DFUIs based on DNA microarray profiling and concluded that nasal *S. aureus* colonization may be a significant DFUI risk factor (Dunyach-Remy et al., 2017). To date, the oral cavity was not investigated and isolates recovered from such sites have not been subjected to WGS. Furthermore, to date, studies of the prevalence and role of other staphylococcal species such as *Staphylococcus epidermidis* in DFUIs are mostly lacking.

The relationship between oral disease and systemic conditions such as cardiovascular disease, bacterial endocarditis and septicemia is well documented (Li et al., 2000). Many studies have reported reciprocal feedback between periopathogens in subgingival plaque and poor glycemic control in patients with diabetes (Atanasova and Yilmaz, 2015; Blasco-Baque et al., 2016) and demonstrated that improved periodontal health benefits glycemic control (D’Aiuto et al., 2018). Additionally, transient bacteremia has been reported in patients with PD following dental scaling or toothbrushing (Forner et al., 2006). We previously revealed that the ubiquitous human commensal and opportunistic pathogen *S. epidermidis* is highly prevalent in the oral cavities (OCs) and PPs of patients with PD, and these isolates predominantly harbor ACME which likely contributes to the success of this species in this anaerobic environment (O’Connor et al., 2018). As PD progresses, staphylococci likely enter the bloodstream through PPs and disseminate, potentially causing metastatic infections. It is also highly possible that these organisms may be transferred from the OC to other anatomical sites by oro-nasal secretions or direct hand transfer.

The objective of this study was to investigate the prevalence and STs of staphylococci from multiple anatomical sites of patients with type 2 diabetes with and without ulcers, to determine if the oro-nasal cavities can act as a microbial reservoir for DFUIs.

## Materials and Methods

### Study Group

Ethical approval for this study was granted by the Tallaght University Hospital and St. James’s Hospital (SJH) Joint Research Ethics Committee. All participants were attending outpatient clinics, >18 years old, capable of providing informed consent and had at least three natural teeth. Patients who were pregnant, lactating, had heart disease or any other underlying diseases were excluded. All participants were sampled during routine outpatient appointments.

### Clinical Sampling Process

At the time of participation, each individual completed a brief survey detailing relevant medical history (Supplementary Table S1).

A 3 cm^2^ area of the skin of the index finger (F) and large toe (T) was swabbed by a qualified podiatrist using separate nitrogen-gassed VI-packed sterile transport swabs (Sarstedt AG & Co.). The podiatrist also assessed and graded the ulcer (U) present as described previously (Lavery et al., 1996). Each U was sampled by the podiatrist by holding a sterile swab in the deepest part of the U for 30 s. All oro-nasal assessments and sampling was undertaken by two calibrated qualified dentists from the Dublin Dental University Hospital (DDUH). Periodontal health was briefly assessed using sterile single periodontal 3-piece examination kits (MDDI, West Yorkshire, United Kingdom). Plaque scores were measured using the Silness and Löe plaque index (Silness and Löe, 1964) and recorded, as were PP depth, bleeding on probing and presence of oral prostheses. Following periodontal examination, the dentist sampled the two deepest PPs using PerioPaper^TM^ gingival fluid collection strips (Oroflow, Plainview, NY, United States), the oral cavity was sampled by oral rinse (OR) and the nares (NS) were swabbed as previously described (O’Connor et al., 2018). All clinical samples were stored between 2 and 8°C during transport to the DDUH microbiology laboratory and processed the same day.

### Microbiological Culture, Isolate Identification and Storage

All samples were processed as previously described, with the F, T, and U swabs being processed in a manner identical to that of the NS swabs (O’Connor et al., 2018). Staphylococci were recovered from samples on Sa*Select*^TM^ chromogenic agar (Bio-Rad Laboratories, Hertfordshire, United Kingdom). Multiple isolates from each sample were definitively identified by matrix-assisted laser desorption/ionization time-of-flight mass spectrometry (MALDI-TOF-MS), routinely cultured and stored for further analysis as previously described (O’Connor et al., 2018).

### DNA Extraction and Molecular Typing

Genomic DNA was extracted from isolates as previously described (O’Connor et al., 2018). Where possible, multiple OR, N, PP, skin, and U isolates of *S. aureus* from each patient were comparatively characterized using the DNA microarray *S. aureus* Genotyping Kit 2.0 [Abbott (Alere Technologies GmbH), Jena, Germany] according to the manufacturer’s instructions. Staphylococcal protein A gene (*spa*) types (Shopsin et al., 1999) were identified using the Ridom StaphyType software version 1.5 (Ridom GmbH, Münster, Germany). Where available, multiple isolates of *S. epidermidis* from distinct anatomical sites of each patient were comparatively characterized using the *S. aureus* Genotyping Kit 2.0 and were subjected to multiplex ACME-typing PCRs as previously described (O’Connor et al., 2018).

### Antimicrobial Susceptibility Testing

The susceptibility of staphylococcal isolates to a panel of 22 antimicrobial agents was determined by disk diffusion according to the European Committee of Antimicrobial Susceptibility Testing methodology and interpretive criteria (European Committee on Antimicrobial Susceptibility Testing, 2017).

### Whole-Genome Sequencing and cgMLST

In order to maximize the discriminatory potential between isolates deemed identical according to conventional typing methods, a total of 22 otherwise indistinguishable *S. aureus* isolates recovered from seven patients with ulcers were sequenced using the Illumina MiSeq short-read sequencing platform (Illumina, Eindhoven, Netherlands). These isolates were selected based on *spa* type, ST and as representatives of multiple distinct anatomical sites from each patient from whom *S. aureus* was recovered.

Libraries were prepared using the Nextera DNA Flex library preparation kit (Illumina) and sequenced using the MiSeq v2 500-cycle reagent kit and a MiSeq desktop sequencer (Illumina) in batches of between 20 and 30 isolate libraries per run. To ensure reliability with downstream epidemiological typing applications, the depth of sequencing coverage was at least 54× per isolate investigated. Following WGS, reads were assembled using Velvet and subjected to cgMLST based on the 1,861 *S. aureus* cgMLST loci previously described (Leopold et al., 2014) using Ridom Seqsphere + version 6.0 (Ridom GmbH). A minimum of 97.3% of cgMLST targets (mean = 98.96%) were present for each isolate investigated. Representative reference genomes of each ST and when possible, *spa* type, were also included in cgMLST analysis for comparison (GenBank). If a reference genome with the same *spa* type was not available, a minimum spanning tree (MST) was constructed based on the cgMLST loci of all the publicly available reference genomes in GenBank with the same ST as the corresponding isolates from this study. The reference genome selected for comparison was that which exhibited the fewest allelic differences to the isolates belonging to the same ST investigated in this study. Ridom Seqsphere + version 6.0 was used to construct MSTs using the default parameters and to confirm *spa* types and STs previously defined by *spa* typing and DNA microarray profiling. All WGS data has been deposited in the NCBI Sequence Read Archive as BioProject PRJNA588375.

## Results

### Patients Investigated

Eighteen patients participated in the study, 13 with foot ulcers. The remaining five patients had no active ulcer or ulcer history. Two of the five patients without ulcers had received antibiotics in the past year and one had received antibiotics and steroids. All five reported brushing their teeth once or twice per day, using interdental cleaning devices and visiting their dentist within the past year. All five patients had oral prostheses such as dentures or a bridge (Supplementary Table S1). Bleeding on probing was observed in one of the five patients and the average plaque score was 58% (range 0–100%). PPs ≥3 mm were detected in all five patients. The average pocket depth sampled was 4.2 mm (range 3–6 mm).

Of the 13 patients with ulcers, nine had received antibiotics in the past year and one had received steroids. All 13 reported brushing their teeth once or twice per day, three of whom also used interdental cleaning devices. Four patients reported attending their dentist within the year prior to participating in this study. Seven of these 13 patients had oral prostheses such as dentures or oral implants. The majority (12/13) of these patients underwent a periodontal examination. Bleeding on probing was observed in 5/12 patients and the average plaque score was 85.8% (range 0–100%). PPs ≥ 3 mm were detected in 11/12 patients with ulcers examined. The average pocket depth recorded was 4.17 mm (range 2–6 mm). The majority (10/13) of patients had type 1A ulcers (superficial wound not involving tendon, capsule or bone) and the remaining three had type OA ulcers (pre- or post-ulcerative lesion completely epithelialized) (Supplementary Table S1).

### Staphylococcal Prevalence

*Staphylococcus aureus* was detected in the nares of 1/5 patients without ulcers (Table 1) and was not detected from other anatomical sites tested in this group. *S. epidermidis* was recovered from the oro-nasal cavities of all five patients, including the OCs of three, and the PPs of two. This species was also recovered from the toe-skin of two patients. Other CoNS were also recovered from the oro-nasal cavities and skin sites of this patient group (Table 1).

**TABLE 1:** Prevalence of staphylococcal species^a^ recovered from each sample site per patient investigated.

**Patient**	**Nasal swab**	**Oral cavity**	**Periodontal pocket**	**Finger**	**Toe**	**Ulcer**
**Patients without foot ulcers**						
G01	SE	SE				NA
G02	SE and CoNS		SE	CoNS	SE and CoNS	NA
G03	SE	SE and CoNS	CoNS		SE	NA
G04	SA and CoNS				CoNS	NA
G05	SE	SE	SE and CoNS		CoNS	NA
**Patients with foot ulcers**						
R01	SE and SA and CoNS	SE and SA		SA and CoNS	SA and CoNS	SA and CoNS
R02	SE and CoNS	SE and CoNS	SE and CoNS	SE and CoNS	CoNS	SE and CoNS
R03	SE and SA	SE	SE and CoNS	SE and SA and CoNS		SA
R04	SA	SE and SA	SE	SE and CoNS	SA and CoNS	SA
R05	SE	SE and CoNS		SE	CoNS	SE and CoNS
R06	SA	SE and CoNS	SE		SA and CoNS	SE and CoNS
R07	SE and SA and CoNS	SA and SE	SA and SE	SE and CoNS	SE and SA	SA and CoNS
R08	SA	SE	SE	SA	SA	SA
R09	SA and CoNS	CoNS	SE and CoNS	SE	CoNS	SE and CoNS
R10	SE		SE	CoNS	SE and CoNS	SE and CoNS
R11	SE and SA and CoNS	SE		SE and CoNS	SE	SA
R12	SE	SE	SE		SA and CoNS	SA
R13	SE and SA		SE	SE and CoNS	CoNS	CoNS

*SE, Staphylococcus epidermidis; SA, Staphylococcus aureus; NA, not applicable; CoNS, coagulase negative staphylococci.*^*a*^*Other coagulase negative staphylococcal species identified: Staphylococcus cohnii, Staphylococcus equorum, Staphylococcus haemolyticus, Staphylococcus saprophyticus, Staphylococcus capitis, Staphylococcus pasteuri, Staphylococcus caprae, Staphylococcus lugdunensis, Staphylococcus hominis, Staphylococcus pettenkoferi, Staphylococcus warneri.*

Thirteen patients with ulcers were sampled. *S. aureus* was more prevalent in this group, recovered from 9/13 NS, 3/13 OR, 1/13 PP, 2/13 F, 6/13 T, and 7/13 U samples (Table 1). Unsurprisingly, *S. epidermidis* was recovered from at least one anatomical site of all 13 patients with ulcers, recovered from 9/13 NS, 10/13 OR, 10/13 PP, 9/13 F, 3/13 T, and 5/13 U samples (Table 1). Other CoNS were isolated from at least one anatomical site of 12/13 patients with ulcers, mostly commonly in combination with *S. aureus*, *S. epidermidis*, or both (Table 1). *S. aureus* was recovered from the ulcers of 7/13 patients, six of whom also yielded *S. aureus* from the oro-nasal cavity (Table 1). Conversely, *S. epidermidis* was detected in the ulcers of a further 5/13 patients, all of whom also yielded this species from the OR or PPs (Table 1).

### Molecular Typing of *S. aureus* Isolates

A total of 49 *S. aureus* isolates recovered from multiple anatomical sites of 10/13 patients with ulcers were subjected to *spa* typing (Table 2). All isolates recovered from distinct anatomical sites were identified as the same *spa* type in 8/10 patients. Two distinct *spa* types were identified in the remaining two patients (Table 2). Isolates recovered from both the oro-nasal and ulcer sites were identified as the same *spa* type in six patients. The predominant *spa* type recovered from distinct patients was t127, detected in isolates recovered from four patients (Table 2).

**TABLE 2:** Clonal Complexes (CCs) and *spa* types identified amongst *S. aureus* isolates recovered from distinct anatomical sites of patients with type 2 diabetes and foot ulcers.

**Patient**	**Isolates^a^ (*n*)**	**Isolation sites (*spa* types identified)**	**CCs^b^ (*n*)**	**Reference genome accession number (ST/*spa* type)**	**cgMLST cluster**	**Branch lengths^c^**	
						**Intracluster average (range)**	**Intercluster^d^**
R01	8	NS (t223), F (t223), T (t223), OR (t223), U (t223)	CC22 (8)	NZ_CP028468 (ST22, t223)	3	0.5 (0–1)	124
R03	6	F (t127), NS (t127), U (t127)	CC1 (5)	NZ_CP013132 (ST1, t127)	5	1.5 (0–3)	190
R04	8	OR (t015), NS (t015), T (t015), U (t015, t127)	CC45 (7), CC1 (1)	NZ_CP017685 (ST45, t015)	2	3.7 (2–6)	303
R06	3	NS (t4802), T (t127)	CC15 (2), CC1 (1)	ND	ND	ND	ND
R07	12	NS (t088), OR (t088), PP (t088), T (t088), U (t088)	CC5 (12)	NZ_LR130541 (ST5, t088)	1	6.7 (3–11)	115
R08	4	NS (t902), T (t852), U (t902)	CC22 (3)	HO 5496 0412 NC_017763 (ST22, t1041)	4	2.5 (1–4)	268
R09	2	NS (t1454)	CC398 (1)	ND	ND	ND	ND
R11	3	NS (t127), U (t127)	CC1 (2)	NZ_CP013132 (ST1, t127)	7	2.5 (0–5)	236
R12	2	T (t008), U (t008)	CC8 (2)	USA300_FPR3757 NC_007793 (ST8, t008)	6	0.5 (0–1)	234
R13	1	NS (t008)	CC8 (2)	ND	ND	ND	ND

*NS, nasal cavity; F, finger-skin; T, toe-skin; OR, oral rinse; PP, periodontal pocket; U, foot ulcer; ND, not determined. ^*a*^The number of isolates indicates the numbers of isolates subjected to spa typing. Isolates selected for further analyses were chosen as representative spa types and distinct anatomical sites of each patient. ^*b*^The clonal complexes (CCs) to which isolates belonged were determined using the S. aureus Genotyping Kit 2.0 (Abbott, Jena, Germany). The number in parentheses (n) indicates the number of isolates subjected to DNA microarray profiling. ^*c*^Branch lengths correspond to the number of allelic differences detected between two adjoining isolates. ^*d*^Inter-cluster allelic differences were defined based on the distances between the founder of each cluster and the most closely related reference genome.*

Forty-six of these 49 *S. aureus* isolates were also investigated by DNA microarray profiling and were assigned to clonal complexes (CCs). These isolates belonged predominantly to CC1 (nine isolates from four patients), CC22 (11 isolates from two patients), and CC5 (12 isolates from one patient). With the exception of isolates from patient R04 which belonged to either CC45 or CC1, all isolates recovered from multiple anatomical sites of each patient were assigned to the same CC (Table 2).

### DNA Microarray Profiling of *S. aureus* From Patients With Ulcers

Forty-six *S. aureus* isolates recovered from patients with ulcers were selected as representatives of distinct patients, sample sites and where possible, distinct *spa* types, were subjected to DNA microarray analysis. The antimicrobial resistance- and virulence factor-encoding genes detected are listed in Supplementary Table S2. No MRSA were recovered (Supplementary Table S2). Phenotypic resistance to ampicillin, fusidic acid, and erythromycin was also detected (Table 3). The previously described L_461_K polymorphism in the ribosomal translocase elongation factor (McLaws et al., 2011) was detected in the WGS data of four CC5 isolates exhibiting fusidic acid resistance. Genes encoding the Panton Valentine leukocidin, exfoliative toxins and epidermal cell differentiation inhibitors, or those associated with ACME were not detected in any *S. aureus* investigated (Supplementary Table S2).

**TABLE 3:** Phenotypic resistance to antibiotics and identification of antimicrobial resistance genes in staphylococcal isolates recovered from patients with foot ulcers.

**Patient**	**Isolates tested (*n*)**	**Phenotypic antimicrobial resistance detected**	**Antimicrobial resistance genes identified^a^**
***S. aureus***			
R01	5	Ap (5)	*blaZ* (5)
R03	3	Fd (3)	*fusC* (3)
R04	4	Ap (4)	*blaZ* (4)
R06	2	Ap (1) Fd (1)	*blaZ, fosB* (1) *fusC* (1)
R07	4	Ap, Fd^b^ (3) Ap, Er, Fd^b^ (1)	*blaZ, fosB* (3) *blaZ, fosB, erm*(C) (1)
R08	3	Ap (3)	*blaZ* (3)
R09	1	Ap, Er (1)	*blaZ* (1)
R11	2	Ap, Er (2)	*blaZ, erm*(C) (2)
R12	2	Ap (2)	*blaZ, fosB* (2)
R13	2	Ap (2)	*blaZ, fosB* (2)
***S. epidermidis***			
R02	5	Susceptible (1) Ap, Er, Fd, Cp, Eb, Fox (1) Ap (1) Ap, Er, Fox, Su^c^ Te (1) Ap, Fd (1)	Negative (1) *blaZ, erm*(C), *dfrS1^d^, fusB, mecA, qacA, qacC* (1) *blaZ* (1) *blaZ, msr*(A), *mph*(C), *mecA, tet*(K) (1) *blaZ, fusB* (1)
R03	2	Kn, Ap, Er, Da, Te, Cp, Cl, Tp, Eb, Fox (1) Ap, Er, Fox, Su^c^ (1)	*blaZ, dfrS1, erm*(C), *aacA-aphD, tet*(K), *cat, qacA* (1) *blaZ, msr*(A), *mecA* (1)
R05	3	Ap, Fd, Su, Tp, Fox (2) Ap, Er, Fd, Su, Tp (1)	*blaZ, dfrS1, fusC, mecA* (2) *blaZ, dfrS1, erm*(C),*fusB* (1)
R06	3	Ap, Fd, Su (1) Ap, Fd (2)	*blaZ, fusC* (1) *blaZ, fusB* (2)
R07	5	Ap, Er, Da, Fd, Te, Cp, Tp, Fox (2) Ap, Fd, Te, Cp, Tp, Fox (1) Ap, Er, Fd (1) Ap, Fd (1)	*blaZ, erm*(C), *dfrS1, fusB, tet*(K), *mecA, qacA^d^* (2) *blaZ, dfrS1, fusB tet*(K), *mecA, qacA^d^* (1) *blaZ, msr*(A), *mph*(C), *fusB, qacA^d^* (1) *blaZ, dfrS1^d^, fusB* (1)
R09	3	Ap, Er (3)	*blaZ, erm*(C) (2) *blaZ, msr*(A), *mph*(C) (1)

*The susceptibility of the staphylococcal isolates to a panel of 22 antimicrobial agents including amikacin (Ak), ampicillin (Ap), cefoxitin (Fox), chloramphenicol (Cl), ciprofloxacin (Cp), clindamycin (Da), erythromycin (Er), ethidium bromide (Eb), fusidic acid (Fd), gentamicin (Gn), kanamycin (Kn), linezolid (Lz), mupirocin (Mp), neomycin (Nm), rifampicin (Rf), spectinomycin (Sp), streptomycin (St), sulphonamide (Su), tetracycline (Te), tobramycin (Tb), trimethoprim (Tp), and vancomycin (Vn). *^*a*^Classes of antimicrobial agents for which the DNA microarray detects resistance genes have been described previously (McManus et al., 2015). ^*b*^The previously described L_461_K polymorphism in the ribosomal translocase elongation factor G (EF-G) was detected in the WGS data for these S. aureus isolates. ^*c*^Intermediate level of resistance exhibited. ^*d*^The dfrS1 gene was detected in two isolates and the qacA gene was detected in four isolates despite the lack of the corresponding resistance phenotypes. Genes encoding resistance to macrolides, lincosamides, and streptogramins were not detected in one S. aureus isolate that exhibited erythromycin resistance*.*

### DNA Microarray Profiling of *S. epidermidis*

Fifty-seven *S. epidermidis* isolates selected as representatives of distinct patients and anatomical sites were subjected to DNA microarray analysis. Fifteen of these isolates were from patients without ulcers and the remaining 42 isolates were from patients with ulcers (Supplementary Table S3).

Antimicrobial resistance-encoding genes were more prevalent in isolates recovered from patients with ulcers (Supplementary Table S3). Multidrug resistance (phenotypic resistance to ≥three classes of clinically relevant antibiotics) was detected in 11 *S. epidermidis* isolates from patients with ulcers (Table 3).

Six of the 15 (40.0%) and 33/42 (78.6%) *S. epidermidis* isolates recovered from patients without and with ulcers harbored ACMEs, respectively. The ACME-positive isolates were recovered from all anatomical sites investigated with the exception of toe-skin, and were identified as ACME types I, II, IV, V, and VI (Supplementary Table S3).

The *ccr* recombinase genes commonly associated with SCC*mec* and SCC elements were detected in 5/15 (33.3%) isolates recovered from patients without ulcers. The SCC*mec* type IV (*n* = 6), the class B *mec* complex (*n* = 6) or other SCC-associated genes were detected in a total of 31/42 (73.8%) isolates recovered from patients with ulcers (Supplementary Table S3). Comparative analyses of DNA microarray profiles, ACME types and antibiotic susceptibility patterns of multiple *S. epidermidis* isolates from each patient revealed that this population was highly diverse. However, multiple *S. epidermidis* isolates recovered from each of eight separate patients were indistinguishable (Supplementary Table S3). In four of these patients, indistinguishable isolates were recovered from distinct anatomical sites (G01: NS and OR; G02: PP and NS; R05: F and OR; R09: F and U).

### Comparison of *S. aureus* Isolates From Distinct Anatomical Sites by cgMLST

Twenty-two *S. aureus* isolates from a variety of distinct anatomical sites of seven patients with ulcers were subjected to WGS and cgMLST. For comparison, six reference genomes representing each of the distinct STs identified among patient isolates were also included in the analysis (Table 3).

**FIGURE 1:**
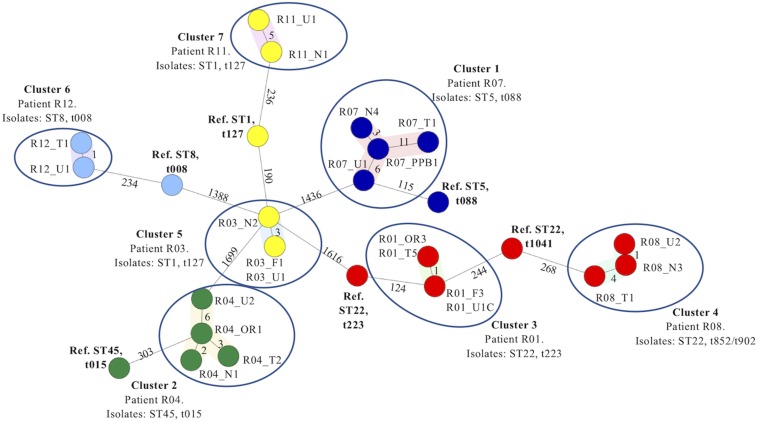
Minimum spanning tree based on the cgMLST analysis of 22 *Staphylococcus aureus* isolates recovered from several anatomical sites in seven patients with type 2 diabetes and ulcers investigated in the present study. Isolates are named according to the patient number and sample site from which each isolate was recovered. Numbers alongside each branch indicate the number of allelic differences between each adjoining isolate. Isolates recovered from the seven patients in each case grouped together into seven distinct clusters (encircled) based on cgMLST analysis (Clusters 1–7). The patient number from whom the isolates were recovered, isolate STs and *spa* types identified are indicated for each cluster, respectively. Six reference genomes recovered from GenBank identified as the same ST (and where possible, *spa* type) of isolates belonging to each cluster (Table 2) were included in the minimum spanning tree as comparators. Abbreviations: F, finger; N, nares; OR, oral rinse; PP, periodontal pocket; Ref, reference genome; ST, sequence type; U, ulcer; T, toe.

Seven distinct clusters of isolates were identified in the MST constructed based on the cgMLST comparison of this dataset (Clusters 1–7, Table 3). Each cluster consisted of a distinct group of isolates recovered from different anatomical sites of each patient investigated (Figure 1 and Table 3). The average branch length corresponded to 2.9 allelic differences (range 0–11). In contrast, the average branch lengths observed between the reference genomes and the most closely related isolate within each cluster was considerably greater (corresponding to an average of 227.1 allelic differences, range 115–303 allelic differences).

Isolates belonging to ST1 and *spa* type t127 were recovered from multiple anatomical sites of both patients R03 and R11. According to the MST, the isolates recovered from each patient grouped into two distinct Clusters, 5 and 7, respectively. No allelic differences were detected amongst the isolates recovered from the finger (R03_F1) and ulcer (R03_U1) and only three allelic differences were detected between these isolates and isolates recovered from the nares (R03_N2) within Cluster 5 (Figure 1). Five allelic differences were detected between the R11_U1 and R11_N1 isolates belonging to Cluster 7. In contrast, isolates belonging to Clusters 5 and 7 exhibited 190 and 235 allelic differences from the reference ST1, t127 genome (NZ_CP013132) respectively (Figure 1 and Table 3).

Isolates identified as ST22 were recovered from several anatomical sites of patients R01 and R08 and identified as *spa* types t223 and t902 or t852, respectively (Table 2 and Figure 1). According to the MST, the isolates recovered from each patient also grouped into two distinct Clusters, 3 and 4, respectively. No allelic differences were detected between isolates R01_OR3 and R01_T5, or between isolates R01_F3 and R01_U1C. Only one allelic difference was detected between these two pairs of isolates within Cluster 3, whereas 124 allelic differences were identified between these isolates and the ST22, t223 isolate reference strain (NZ_CP028468). One allelic difference was detected between R08_U1 and R08_N3 and four allelic differences were detected between R08_N3 and R08_T1. Due to the lack of a publicly available ST22, t015 reference genome, the most appropriate ST22 reference genome was selected due to its closest relationship to the isolates in Cluster 4 (NO 0596 0412 NC_017763) based on a separate cgMLST analysis of publicly available reference genomes for ST1 isolates only. There were 268 allelic differences detected between R08_T1 and the ST22, t1041 isolate reference strain (HO 5496 0412 NC_017763).

Isolates recovered from multiple anatomical sites of patients R04, R11 and R12 were identified as distinct ST/*spa* types, ST45/t015, ST1/t127 and ST8/t008, respectively. The MST based on cgMLST grouped these isolates into three distinct Clusters 2, 7 and 6, respectively (Figure 1). The maximum branch length within each of these clusters was six, whereas branch lengths between each cluster and the appropriate reference genome was 303, 236, and 354, respectively.

Together, these datasets revealed that in each of the seven clusters, isolates recovered from multiple distinct anatomical sites of the same individual were highly related or genetically indistinguishable by cgMLST, in contrast to isolates recovered separately, despite being identified as the same ST and *spa* type (Figure 1 and Table 3).

## Discussion

The development of a DFUI can be catastrophic, significantly increasing the risk of foot amputation which can have life-changing consequences for patients, their families and place significant additional demands on the healthcare system. The incidences of diabetes-related lower limb amputations are continually increasing in Ireland (Diabetes Ireland, 2015).

Recent research has suggested that periodontal treatment can improve the glycemic indices of patients with diabetes (Casanova et al., 2014), and bacterial entry into the bloodstream during non-invasive oral hygiene processes has been demonstrated previously (Forner et al., 2006). To date, there has been no comprehensive examination of the role of the oro-nasal and periodontal tissues as endogenous sources for DFUIs. The identification of such reservoirs could be highly informative and beneficial in the design of preventive interventions such as oro-nasal decolonization and periodontal treatments. To our knowledge, this is the first study to investigate the periodontal tissues and oral cavity as a potential endogenous source of DFUI, including the application of WGS to investigate the genetic relationship between *S. aureus* isolates recovered from the oro-nasal cavity and DFUIs of patients with diabetes.

*Staphylococcus aureus* was considerably more prevalent in the patients with ulcers investigated (Table 1). Of the 13 patients with ulcers investigated, *S. aureus* was recovered from seven ulcers, *S. epidermidis* was recovered from a further five and the remaining ulcer yielded other CoNS only. This data suggests that *S. epidermidis* may also play an important role in DFUI etiology. As *S. epidermidis* is a proficient biofilm former, an opportunistic pathogen and commonly exhibits multidrug resistance, its common presence at these sites should not be overlooked. Six of the seven patients from whom *S. aureus* was recovered from ulcer sites also yielded *S. aureus* from oro-nasal sites. Similarly, all six patients from whom *S. epidermidis* was recovered from ulcer sites also yielded the same species from the OR or PPs.

Unsurprisingly, genes encoding antibiotic resistance were more prevalent in isolates recovered from patients with ulcers, most likely reflecting previous antibiotic treatment (Supplementary Table S1). Multidrug resistance was common in *S. epidermidis* isolates recovered from these patients, illustrating the higher prevalence of antimicrobial resistance encoding genes and SCC*mec*-associated genes reported in this species generally (Otto, 2013; McManus et al., 2015). DNA microarray profiling and analysis of ACME types and antibiotic susceptibility patterns revealed a highly diverse population of *S. epidermidis* isolates in each participant investigated (Supplementary Table S3), correlating with our previous research (O’Connor et al., 2018). Multiple *S. epidermidis* isolates recovered from distinct anatomical sites were indistinguishable in four patients (Supplementary Table S3), however, indistinguishable isolates were not recovered from the ulcer or oro-nasal cavity in any patient investigated. These datasets indicate that diverse *S. epidermidis* populations exist both within each anatomical site and between distinct anatomical sites. Future studies should investigate larger numbers of isolates from each anatomical site per patient to determine if *S. epidermidis* populations amongst each distinct anatomical site are similar.

The application of WGS has significantly enhanced the resolution, sensitivity and scale under which global bacterial surveillance and comparative analyses can now be performed. Several practical bioinformatic tools have been developed to aid the routine application of *S. aureus*-based WGS technologies in clinical microbiology laboratories, such as highly discriminatory strain comparison for outbreak and transmission investigations and rapid prediction of antimicrobial resistance patterns. Such software packages are largely responsible for the increased application of WGS in healthcare settings where bioinformatic support is lacking (Leopold et al., 2014; Ruan and Feng, 2016; Deurenberg et al., 2017).

Conventional molecular typing tools suggested that *S. aureus* isolates recovered from oro-nasal and ulcers sites were highly related, being identified as the same ST or *spa* type in 8/10 patients with ulcers from whom this species was recovered (Table 2), correlating with previous studies suggesting nasal colonization as a DFUI risk factor (Stanaway et al., 2007; Dunyach-Remy et al., 2017; Lin et al., 2018). The investigation of 22 of these isolates from several anatomical sites in seven patients with ulcers by WGS and subsequent cgMLST confirmed this. In each patient, the oro-nasal and DFU isolates recovered differed by a maximum branch length corresponding to 11 allelic differences (Figure 1), less than half of the ≤24 allelic difference threshold for relatedness recently suggested for *S. aureus* (Schürch et al., 2018). In contrast, the numbers of allelic differences between isolates investigated in the present study and previously sequenced reference genomes ranged between 115 and 354 allelic differences (Figure 1 and Table 3), despite being selected on the basis of the same *spa* type and/or ST. Together, these datasets provide irrefutable evidence that isolates recovered from the oro-nasal cavity and ulcers in the same patient are the same strain.

The principal and secondary *S. aureus* colonization sites include the nares, hands, perineum, oro-pharynx, and axillae. This species rarely colonizes the feet (Wertheim et al., 2005). Previous research has suggested the nares as a potential endogenous source of *S. aureus-*based DFUIs (Stanaway et al., 2007; Dunyach-Remy et al., 2017; Lin et al., 2018) and our previous research revealed that both *S. epidermidis* and *S. aureus* are prevalent in the mouths of patients with periodontal disease (O’Connor et al., 2018). It is also known that periodontal disease is more prevalent and more severe in patients with diabetes (Casanova et al., 2014). Two separate studies reported that individuals touch their own face with their hands an average of 23 times per hour (Kwok et al., 2015). The facial sites most commonly touched were the mouth and nose (Elder et al., 2014). In this context, it is likely that resident oro-nasal staphylococcal bacteria may be transferred to the feet by direct hand-transfer following facial self-touch. Bacterial entry to the bloodstream through PPs before being transported to other suitable niches such as foot ulcers is also possible. In combination, these research findings suggest how periodontal disease and oro-nasal carriage likely play an important role in diabetic foot health and has important implications for infection prevention and control strategies, both in healthcare settings and in self-minimization of endogenous infection risk.

One obvious limitation of the present study is the small number of patients investigated, however, each patient was sampled at a minimum of four anatomical sites and multiple isolates of all staphylococcal species recovered were identified presumptively by colony morphology on chromogenic media and/or by MALDI-TOF-MS. The primary objective of this preliminary study was to compare the staphylococcal species in the oro-nasal and DFU sites of patients with diabetes. In addition, *S. aureus* isolates recovered from both oro-nasal and DFU sites of multiple distinct patients were directly compared using WGS for the first time. Further studies are required to identify additional links between oro-nasal carriage, periodontal health and DFU status, including data on glycemic indices, presence and severity of periodontal disease and DFUIs and detailed antibiotic usage history. Such investigations should include a larger group of patients, WGS analysis of a larger numbers of both *S. aureus* and *S. epidermidis* isolates per sample as well as per patient, and more in-depth investigation of isolate relatedness using WGS data. Such investigations are ongoing.

## Data Availability Statement

The datasets generated for this study can be found in the NCBI Sequence Read Archive: BioProject PRJNA588375.

## Ethics Statement

The studies involving human participants were reviewed and approved by Tallaght University Hospital and St. James’s Hospital Joint Research Ethics Committee. The patients/participants provided their written informed consent to participate in this study.

## Author Contributions

BM conceived and designed the study, assisted with sample collection, performed WGS, analyzed data, and prepared the manuscript. BD, IP, M-LH, and PW designed the study, assisted with data collection, and reviewed the manuscript. GB, TF, and LG assisted with data analysis and reviewed the manuscript. DC designed the study, purchased the required materials, assisted with data analysis, and reviewed the manuscript.

## Conflict of Interest

The authors declare that the research was conducted in the absence of any commercial or financial relationships that could be construed as a potential conflict of interest.
